# The Contribution of the Amyloid Hypothesis to the Understanding of Alzheimer's Disease: A Critical Overview

**DOI:** 10.1155/2012/709613

**Published:** 2012-08-28

**Authors:** Laura Morelli, George Perry, Fabrizio Tagliavini

**Affiliations:** ^1^Laboratory of Amyloidosis and Neurodegeneration, Fundación Instituto Leloir, IIBBA-CONICET, C1405BWE Buenos Aires, Argentina; ^2^University of Texas at San Antonio, San Antonio, TX, USA; ^3^Department of Neurodegenerative Diseases, Carlo Besta Neurological Institute (CBNI), Milan, Italy

This special issue is devoted to revisit the “amyloid cascade hypothesis” (ACH) in the pathogenesis of sporadic Alzheimer's disease (AD). Since the identification 20 years ago of the first APP mutation [1] the ACH gained enormous importance based on genetic and biochemical evidence. However the outcome of recent clinical trials aimed at reducing extracellular A*β* levels suggests that such strategy may not have the expected impact on AD progression because the role of A*β* is more complex than that of the lone driver of AD. Some of the reasons proposed for the failure may be the initiation of the trials in demented patients with serious brain damage, and unforeseen serious design flaws in the studies. These results led us to ask whether A*β* plays an active protective role in brain aging. It is also clear that regardless of whether A*β* is protective or toxic, trials focused on modulating the A*β* response will remain a major interest in AD therapeutic research.

According to the amyloid cascade hypothesis, increased amounts of A*β* contribute to the development of AD [2]. A*β* peptides are generated in the amyloidogenic pathway of APP processing by sequential proteolysis by *β*- and *γ*-secretases. In the alternative nonamyloidogenic APP processing pathway, *α*-secretase cleaves within the A*β* peptide region and prevents A*β* generation. Increasing the *α*-secretase-mediated processing of APP may therefore be a therapeutic option for the treatment of AD. Since various substrates have been assigned to *α*-secretase-like cleavage events, putative side effects of *α*-secretase activators should be considered. BACE1, the catalytic component of *β*-secretase, is the key enzyme initiating A*β* production *in vivo*, making it a prime drug target for AD treatment. The past decade has shown significant progress in the understanding of BACE1 molecular and cellular properties, however, further investigation is crucial to predict side effects of BACE1 inhibition. *γ*-Secretase complex represents a fascinating biological machine that is assembled from at least four core proteins (presenilins 1 or 2, APH1, PEN2, and nicastrin). These proteins are sufficient for cleavage of multiple different, nonhomologous type 1 transmembrane (TM) proteins, with the cleavage occurring through the substrates' TM domains. *γ*-Secretase remains a target of intense interest for modulating A*β*. Nowadays, the focus has clearly shifted toward modulators that minimize effects on other substrates (in particular notch), with compounds that either shift the site of cleavage to produce shorter forms of A*β* or selectively inhibit APP processing while allowing the enzyme to continue processing notch. Compounds now under investigation may not have sufficient potency, brain penetration, or selectivity to effectively lower brain A*β* while avoiding notch-related toxicity. Recently, another secretase-mediated APP-derived catabolite called APP Intra Cellular Domain (AICD) gained relevance in the field appears to be a multifunctional factor affecting several physiological processes likely to contribute to Alzheimer's disease pathology by acting as a transcription factor that controls the expression of a series of proteins involved in control of cell death and A*β* degradation.

The steady state of monomeric A*β* in the brain is the result of a tightly controlled balance between production and removal; sporadic AD may reflect defects in clearance mechanisms for A*β* rather than in the enhanced synthesis which occurs in early-onset cases. It was recently demonstrated that the kinetics of A*β* production is similar between control and late-onset AD patients, however there is an impairment in the clearance of A*β* in AD as compared to controls, indicating that A*β* clearance mechanisms may be critically important in AD [3]. Among these mechanisms, interaction of A*β* with ApoE, decreased catabolism via reduced proteolysis, impaired transport across the blood-brain barrier, and impaired CSF transport deserve special attention. Based on experimental evidence in animal models of AD, upregulation of amyloid degrading enzymes (ADEs) individually in the brain appears to be a viable strategy to reduce the amyloid burden and improve cognitive function. However, these animal models in themselves have limitations to representing the human disease. 

With evidence that the extent of insoluble, deposited amyloid poorly correlated with cognitive impairment, research efforts focused on soluble forms of A*β*, also referred to as A*β* oligomers. Following a decade of studies, soluble oligomeric forms of A*β* are now believed to be the most biologically active form of A*β*. Understanding the events triggered by oligomeric A*β* species has greatly improved in the past years but specific efforts are required to understand the molecular mechanism(s) of endogenous A*β* assemblies. Brain amyloid deposits contain proteins besides A*β*, such as apolipoprotein E (apoE). Significantly, inheritance of the apoE4 allele is the strongest genetic risk factor for the most common, late-onset form of AD. However, there is no consensus on how different apoE isotypes contribute to AD pathogenesis. It has been hypothesized that apoE4 in particular is an amyloid catalyst or “pathological chaperone”. Evidence from numerous epidemiological studies indicates that type 2 diabetes, a non-insulin-dependent form of diabetes mellitus, is associated with a 2- to 3-fold increase in the relative risk for sporadic AD. Experimental evidence suggests that abnormalities in insulin metabolism in diabetic conditions could mechanistically influence the onset of AD via modulation of the synthesis and degradation of amyloidogenic A*β* peptides, providing a molecular link between metabolic dysfunction and neurodegenerative process in the elder population.

In this special issue D. A. Bórquez and C. González-Billault review the potential role of multiprotein complexes between the AICD and its adapter protein Fe65 and how these complexes impact on the neurodegeneration observed in AD. G. M. Pasinetti and colleagues describe the role of insulin receptor (IR) signaling mechanisms in the onset and/or progression of AD dementia and the relevance of insulin-sensitizing therapeutic strategies to stimulate down-stream IR in nondiabetic AD patients. C. Reitz critically reviews the evidence for and against the amyloid cascade hypothesis in AD and provides suggestions for future directions. T. Wisniewski and Huntington Potter consider the scientific basis of the contrasting views of apoE's role, suggesting that these seemingly opposing views can be reconciled. A. J. Turner and colleagues critically evaluate general biochemical and physiological functions of Neprilysin, one of the relevant ADEs in the human brain, and their therapeutic relevance.

We hope that this focused series of articles will provide the readers a critical overview of current understanding of A*β* deposition in AD.

*Laura Morelli* *Laura Morelli* *George Perry* *George Perry* *Fabrizio Tagliavini* *Fabrizio Tagliavini*
